# Supplementary material for: Unraveling the Roles of Epigenetic Regulators During the Embryonic Development of Rhipicephalus microplus
Source: Int J Mol Sci. 2025 Sep 19;26(18):9171. doi: 10.3390/ijms26189171 (PMC12470784; doi:10.3390/ijms26189171)
Supplement: Supplementary file 1 [file ijms-26-09171-s001.zip › Supplementary Figure S1.pdf]

A

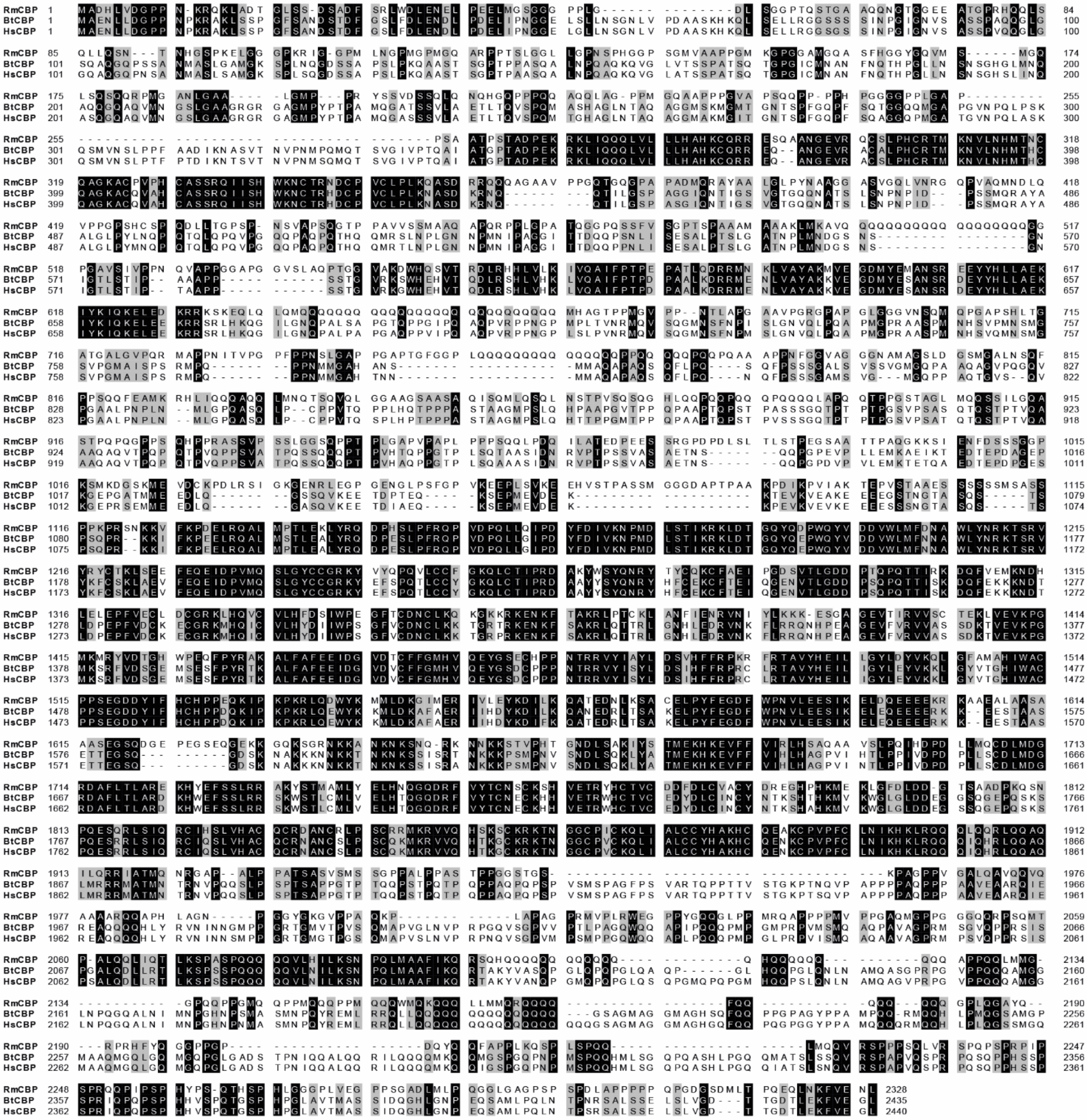

B

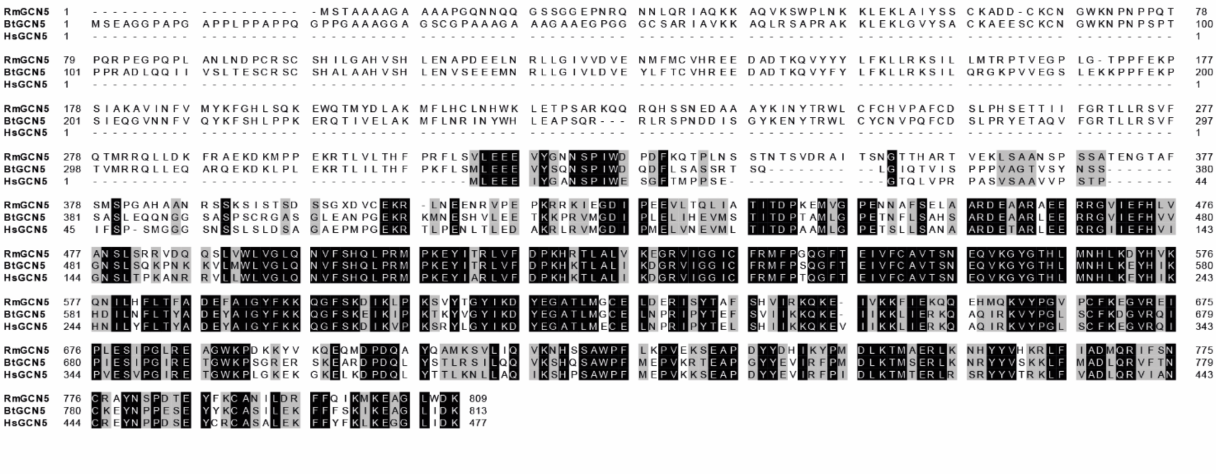

C

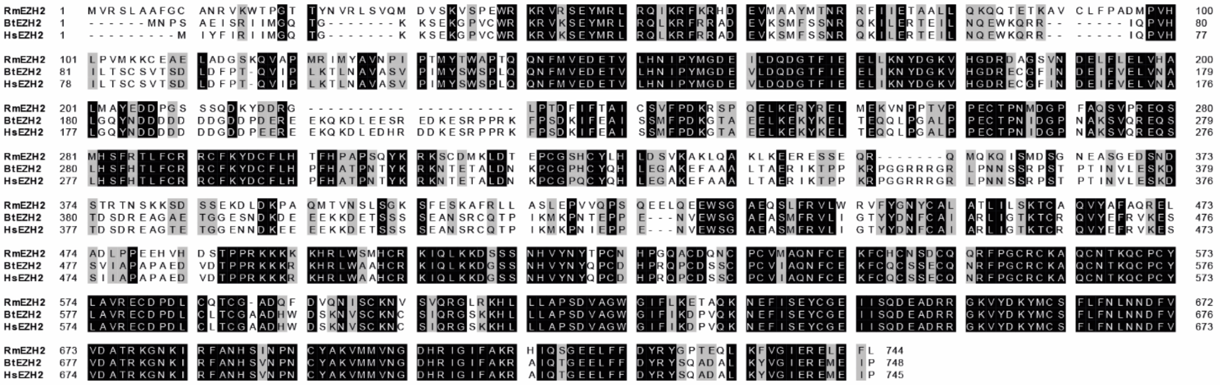

D

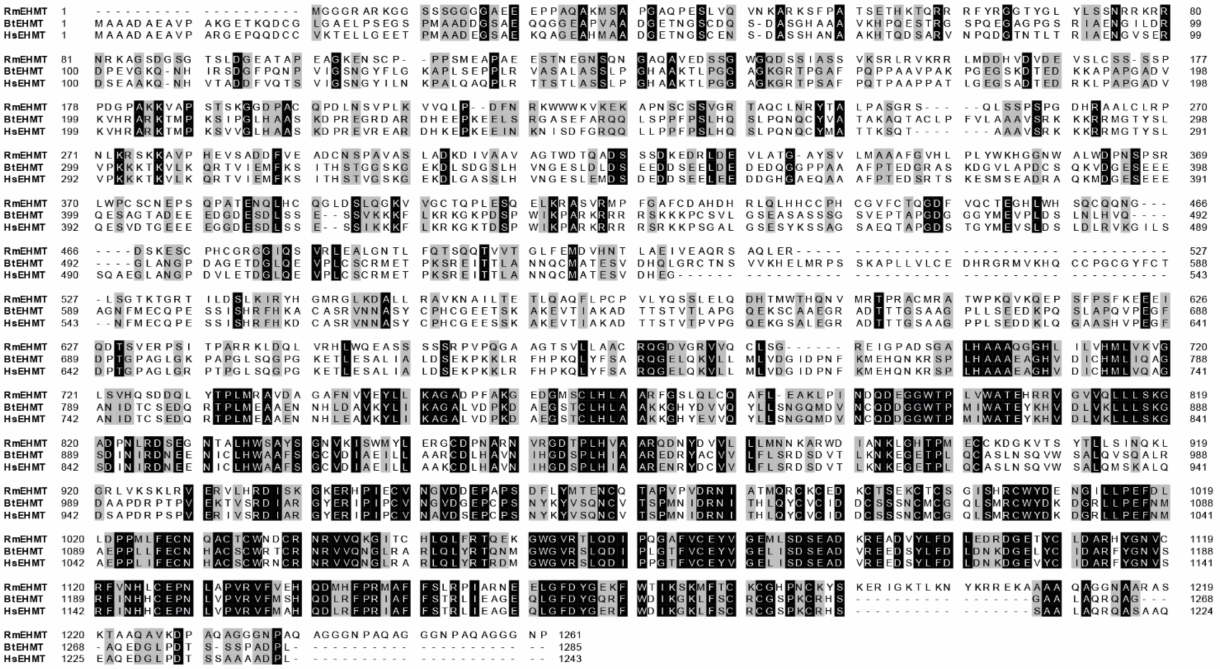

E

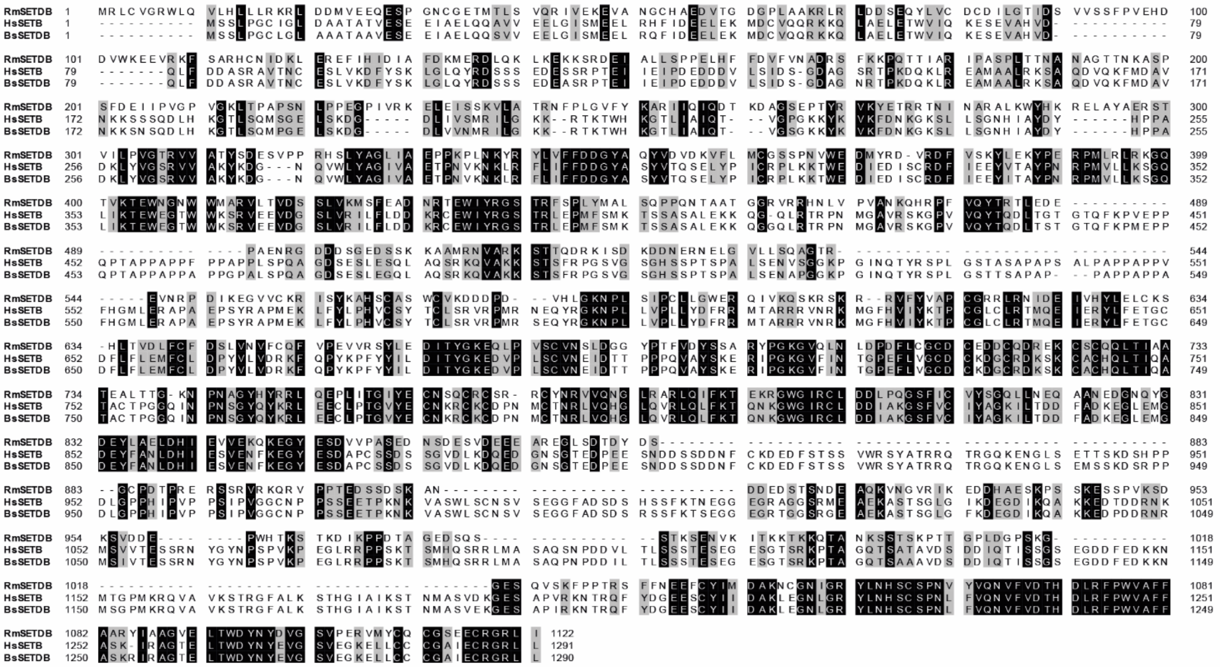

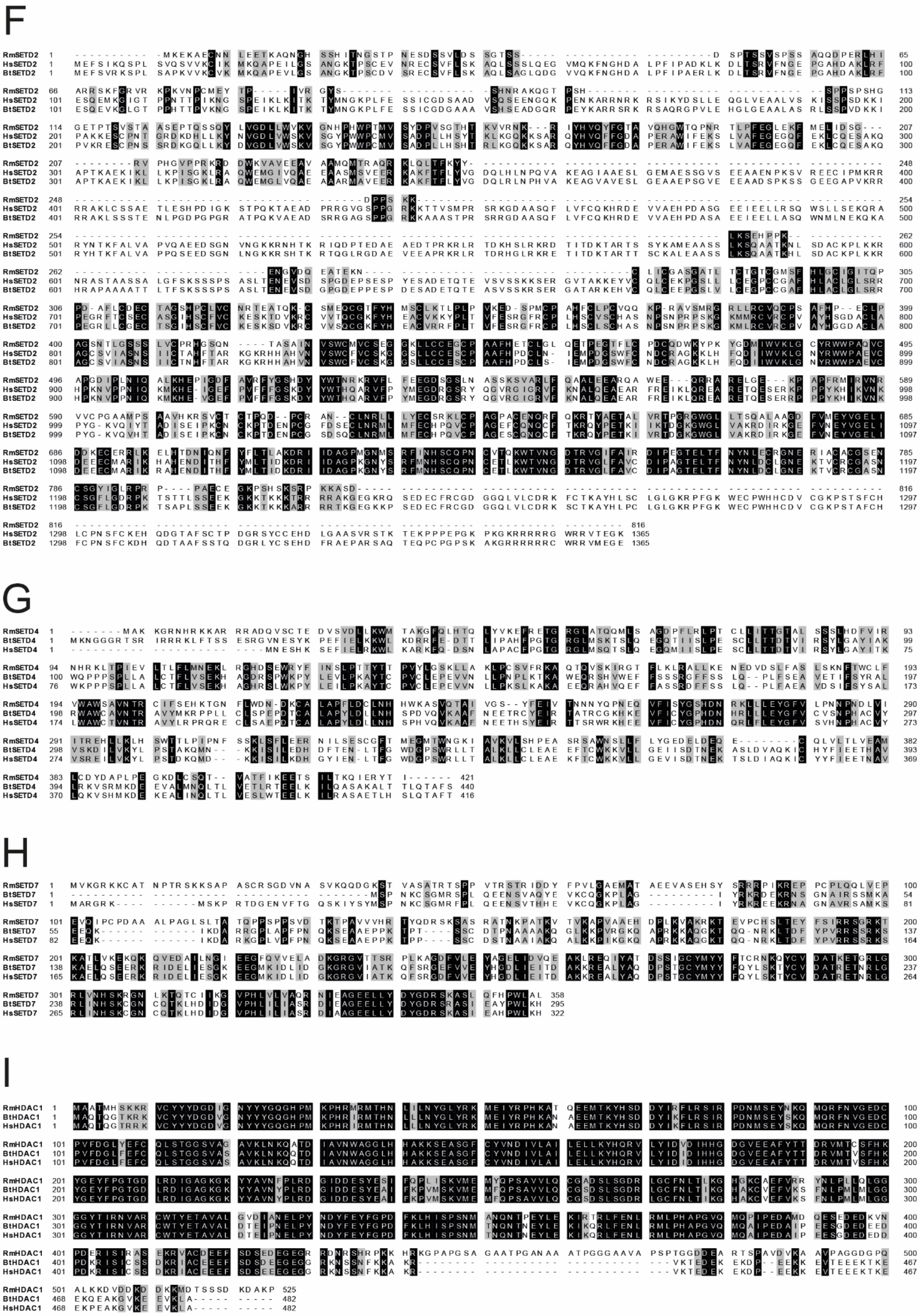
F
RmSETD2
HsSETD2
BtSETD2
G
RmSETD4
BtSETD4
HsSETD4
H
RmSETD7
BtSETD7
HsSETD7
I
RmHDAC1
BtHDAC1
HsHDAC1

J

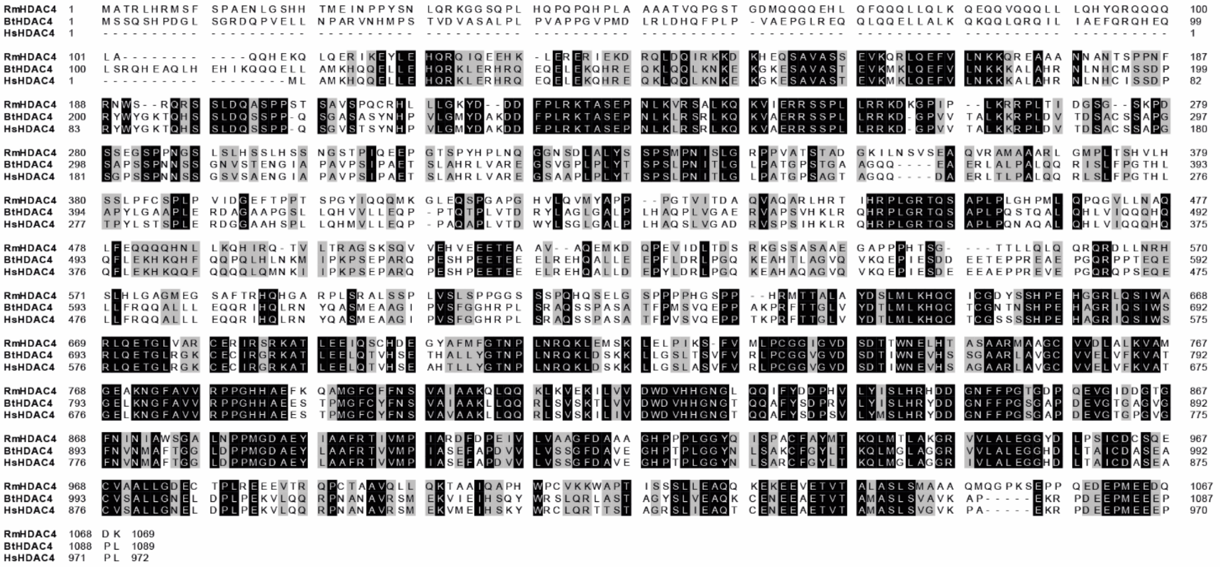

K

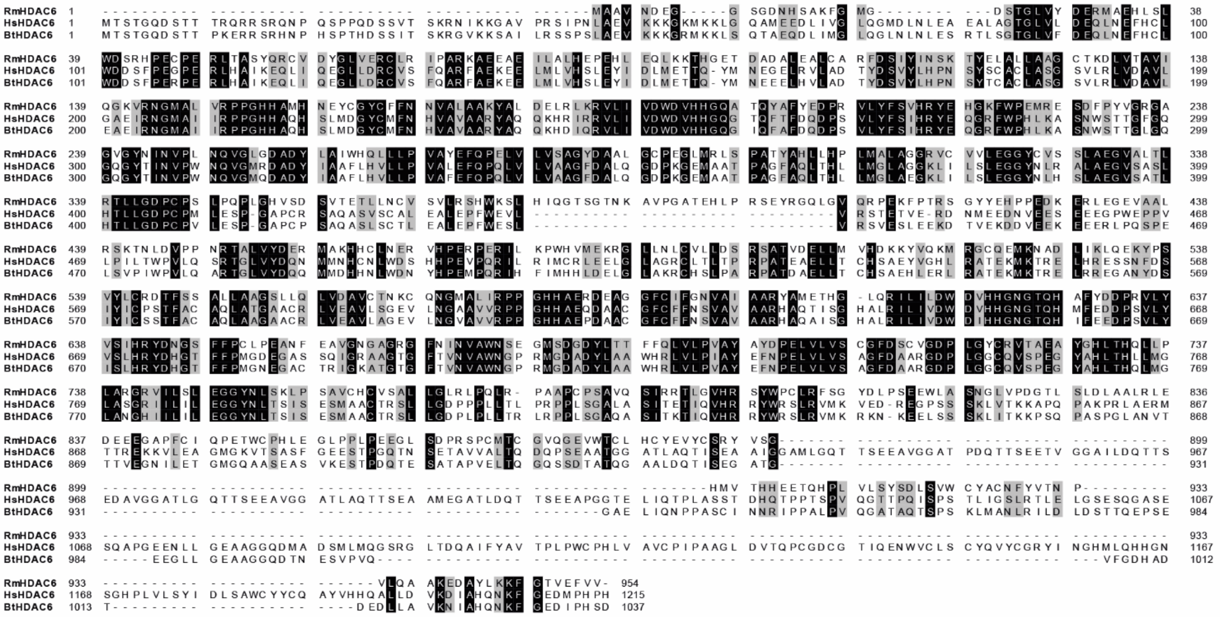

L

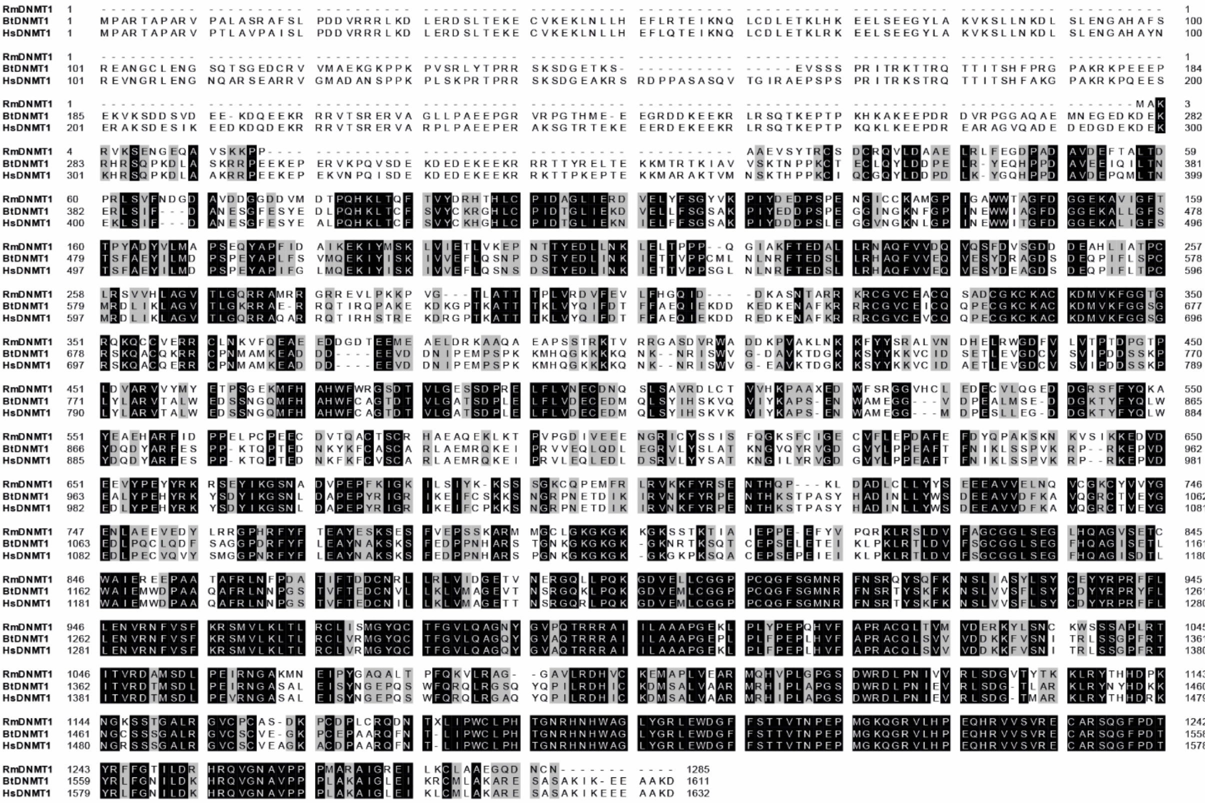

M

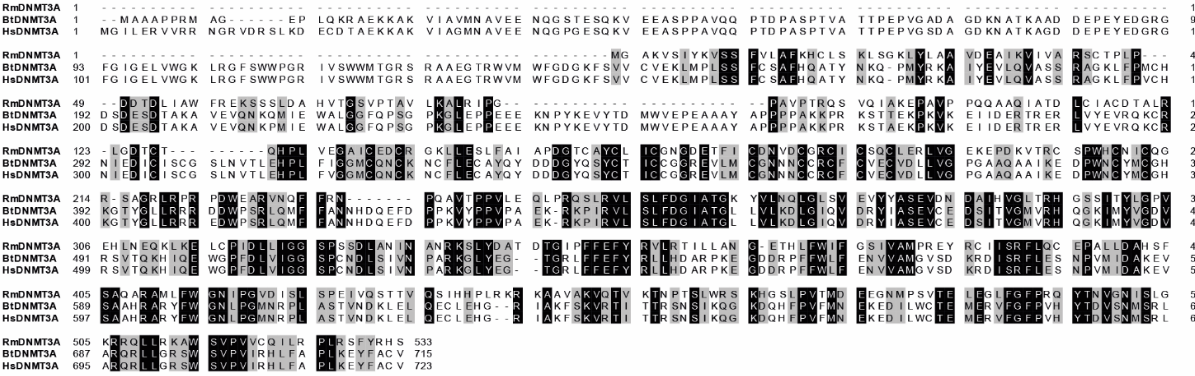

N

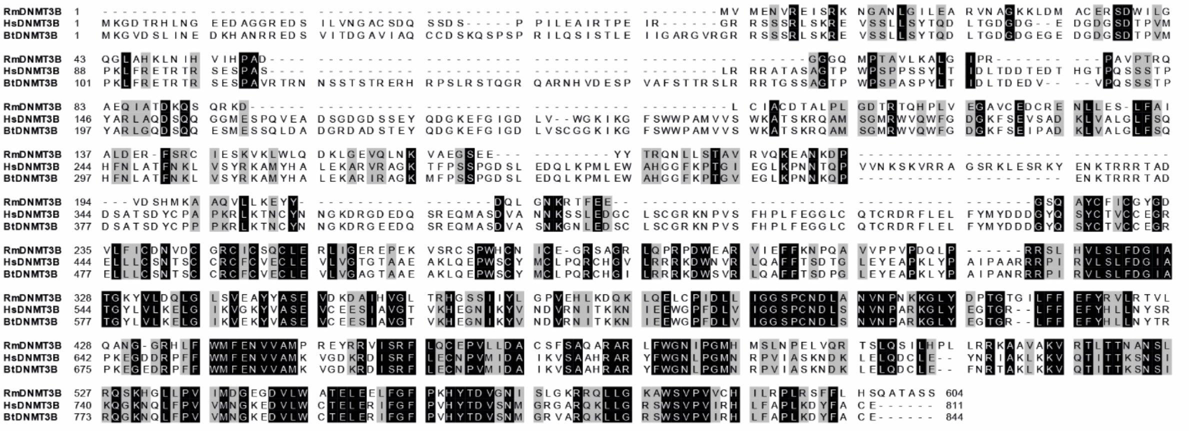

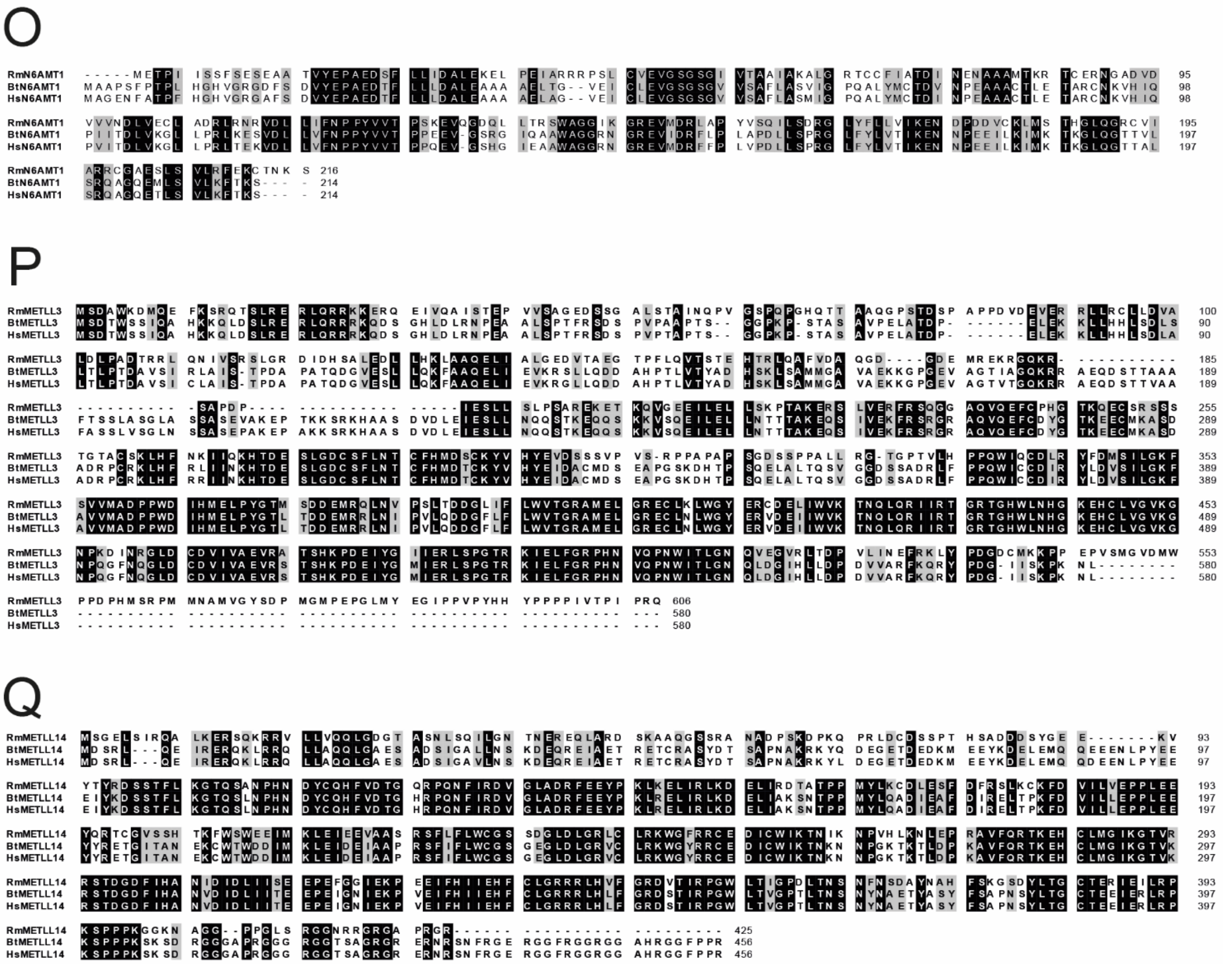

Supplementary Figure S1. Protein sequence alignment of full-length epigenetic regulators from *Rhipicephalus microplus* (Rm), human (Hs), and bovine (Bt). Amino acids highlighted in gray represent conserved residues, whereas those highlighted in black indicate identical residues across species.
