# Supplementary material for: Unraveling the Roles of Epigenetic Regulators During the Embryonic Development of Rhipicephalus microplus
Source: Int J Mol Sci. 2025 Sep 19;26(18):9171. doi: 10.3390/ijms26189171 (PMC12470784; doi:10.3390/ijms26189171)
Supplement: Supplementary file 1 [file ijms-26-09171-s001.zip › Supplementary Figure S2.pdf]

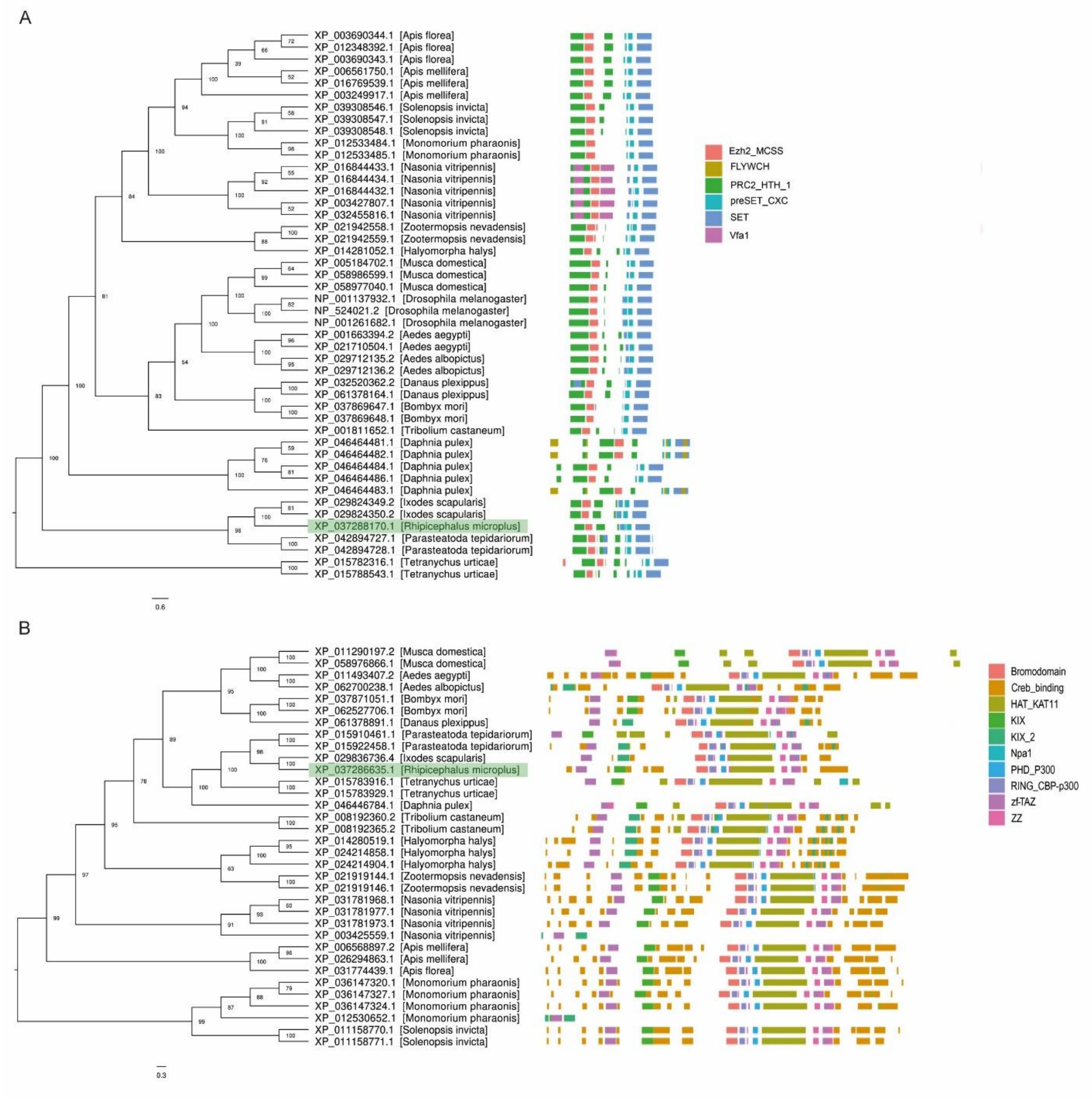

Supplementary Figure S2. (A,B). Phylogenetic trees using Ezh and CBP-p300 orthologs across different species, with domain architecture representation. The tree on the left represents evolutionary relationships based on sequence similarity. Bootstrap support values are indicated at each node. On the right, colored bars represent conserved protein domains, as annotated in the legend. The highlighted sequence from *Rhipicephalus microplus* indicates our species of interest. Domain annotations are based on Pfam.

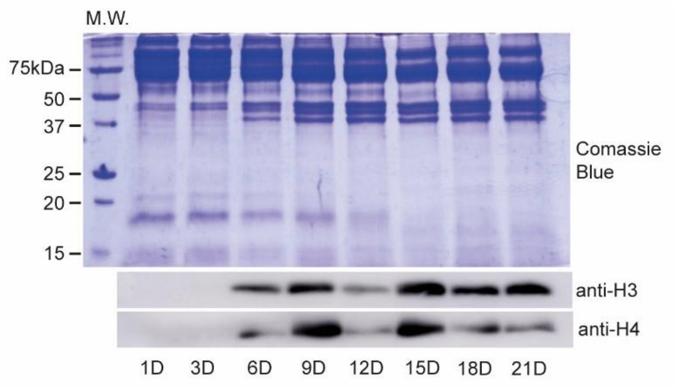

Supplemental Figure 3. SDS-PAGE and Western blot analysis of total protein extracts from Rhipicephalus microplus eggs. Eggs from Days 1 (1D) to 21 (21D) were probed for the presence of histones H3 and H4 using monoclonal antibodies. Histone levels are notably absent at Days 1 and 3, as indicated by the lack of corresponding bands

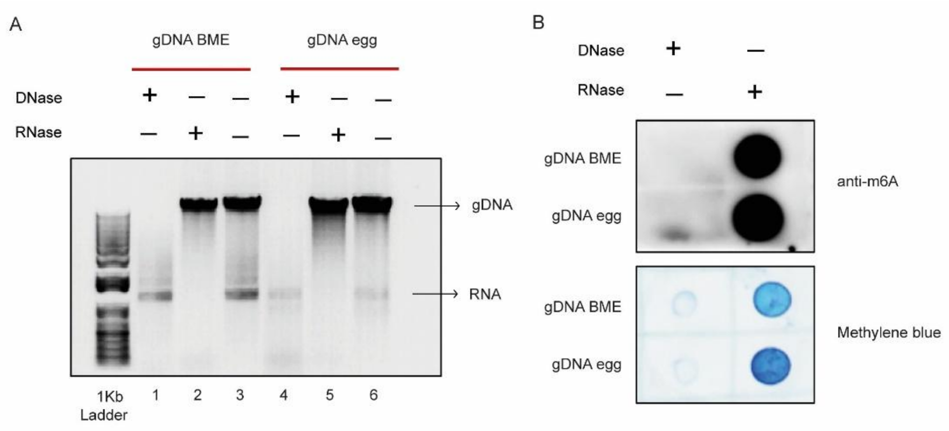

Supplemental Figure 4. Detection of m⁶A methylation in genomic DNA of *Rhipicephalus microplus*. (A) Agarose gel electrophoresis of genomic DNA isolated from BME26 cells and eggs. The upper band (arrow) corresponds to genomic DNA, whereas the lower band (arrow) indicates RNA contamination. Treatment with RNase A effectively removed RNA contaminants (Lanes 3 and 6). (B) Genomic DNA from cells or eggs that were treated with RNase A was evaluated by dot blot analysis which revealed the presence of m⁶A methylation in RNA-free genomic DNA.

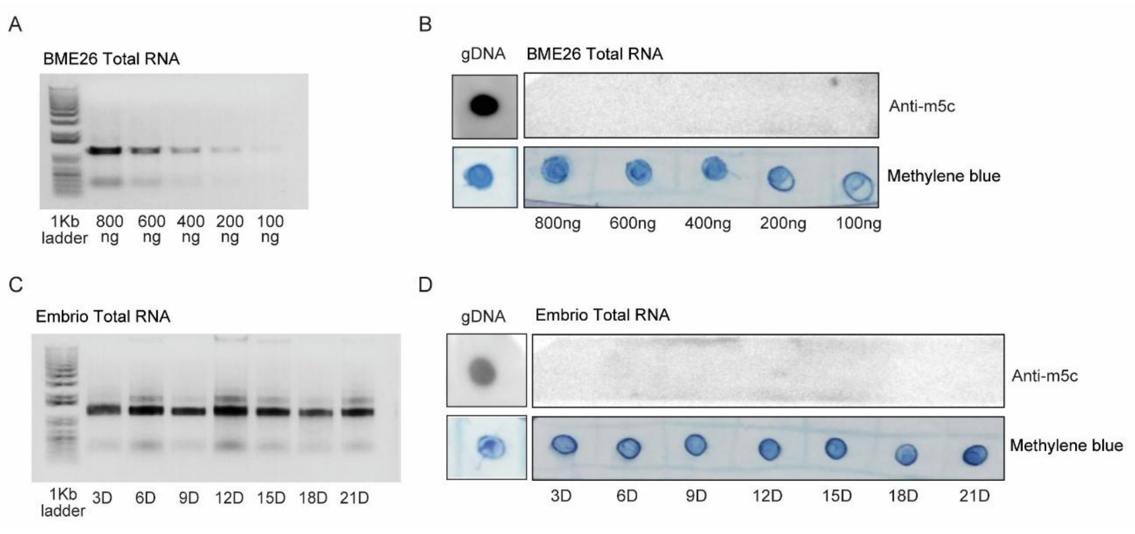

Supplemental Figure 5. Lack of 5-methylcytosine (m^5C) modification in samples of total RNA isolated from *Rhipicephalus microplus*. (A, C) Agarose gel electrophoresis of total RNA isolated from BME26 cells (A) and embryos collected from Day 3 to Day 21 (C), confirming RNA integrity. (B, D) Dot blot analysis showing no detectable m^5C methylation in RNA from BME26 cells (B) or embryos (D). Genomic DNA from the same samples was used as a positive control, demonstrating the presence of m^5C DNA methylation (gDNA).

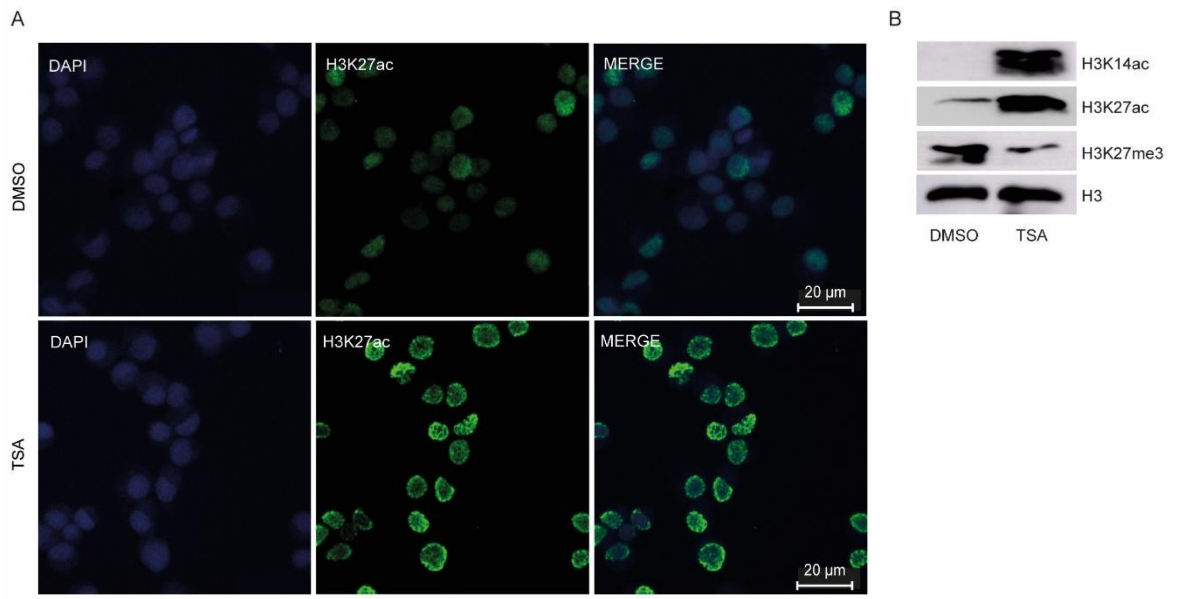

Supplemental Figure 6. Hyperacetylation of histones in BME26 cells. (A) BME26 cells were treated with 100 µM TSA for 48 hours followed by immunostaining with an anti-H3K27ac monoclonal antibody (green) and nuclear counterstaining with DAPI (blue). Confocal microscopy revealed a significantly greater H3K27 acetylation than in the control, confirming the effective inhibition of HDAC activity. Scale bar: 10 µm. (B). Western blot analysis of histone modifications in BME26 cells (as shown in Panel A). Protein extracts were analyzed for specific histone modifications to assess epigenetic changes. Increased levels of acetylation at histone H3 lysine 14 (H3K14ac) and lysine 27 (H3K27ac) indicate hyperacetylation in response to the experimental conditions. In contrast, a reduction in trimethylation at H3K27 (H3K27me3) was detected, supporting the antagonistic relationship between acetylation and methylation at this residue.

Table 1. List of antibodies used in this study

Primary antibodies	Code
Anti-H3, Cell Signaling	14269
Anti-H3K27ac, Cell Signaling	8173
Anti-H3K14ac, Cell Signaling	7627
Anti-H3K4me3, Cell Signaling	9727
Anti-H3K9me2, Cell Signaling	4658
Anti-H3K9me3, Cell Signaling	13969
Anti-H3K27me3, Cell Signaling	9733
Anti-m6A, Invitrogen	MA5-35350
Anti-m5C, Abcam	ab214727
**Secundary antibodies**	**Code**
Anti-mouse, Invitrogen	31430
Anti-Rabbit, Invitrogen	32460
Anti-Rabbit Alexa fluor 488, Invitrogen	A11001

Table 2. Protein sequence homology of epigenetic regulators across Rhipicephalus microplus, Bos taurus, and Homo sapiens. GenBank accession numbers are listed for each protein, along with the percentage identity of full-length sequences between R. microplus and the other species (B. taurus and H. sapiens). Sequence alignments were performed using [tool/algorithm, e.g., BLASTP] with default parameters.

Protein name	*R. micropoulos*	*B. taurus*		*H. sapiens*	
	GenBank ID	GenBank ID	Identity	GenBank ID	Identity
CBP/P300	XP_037286635.1	NP_001157494.1	61%	XP_016878433.1	61%
EZH2	XP_037288170.1	XP_059741477.	59%	XP_005250020.1	58%
EHMT	XP_037291703.1	XP_024854576.1	52%	XP_054219807.1	50%
SETDB1-A	XP_037277427.1	NP_001178317.1	46%	NP_001380890.1	45%
SETD2	XP_037282969.1	XP_024849516.1	49%	NP_001035889.1	49%
SETD4	XP_037281861.1	NP_001192753.1	32%	NP_001273681.1	34%
SETD7	XP_037279979.1	XP_024833097.1	47%	NP_001311433.1	52%
GCN5	XP_037291980.1	XP_024853358.2	63%	XP_006721881.1	60%
HDAC1	XP_037271577.1	NP_001032521.1	87%	NP_004955.2	85%
HDAC4	XP_037291540.1	XP_059741238.1	47%	AAH39904.1	47%
HDAC6	XP_037286423.1	XP_024843702.1	45%	AAH69243.1	47%
DNMT1	XP_037279276.1	XP_024849600.1	57%	NP_001124295.1	57%
DNMT3A	XP_037270226.1	XP_059747024.1	39%	NP_715640.2	39%
DNMT3B	XP_037270264.1	XP_024856107.1	47%	NP_001411281.1	47%
N6AMT1	XP_037277348.1	NP_001076982.1	48%	NP_037372.4	50%
METLL3	XP_037282032.1	XP_024853529.1	50%	NP_062826.2	54%
METLL14	XP_037281356.1	NP_001077183.1	64%	NP_066012.1	64%

Table 3. Sequences of primers used in this study. GenBank identification is provided for all genes.

Protein name	GenBank ID	Primer Foward	Primer Reverse
RmP300/CBP	XM_037430738.1	AAGCCATGTGGAGACACGTT	TCGAGGTCAAAGCCCAACTT
RmEZH2	XM_037432273.1	ATTGGTGCTACCTGCTTGCT	ACGCTTCAAAAGGCATGACG
RmEHMT	XM_037435806.1	AAACATCCCAGCAAACCGTTG	TGTTTTGGCTGTCCTATTGCTG
RmSETDB1-A	XM_037421530.1	AAACGTCTGCGCTTGGATGA	GAACATTTGCTCAACCGGGAAG
RmSETD2	XM_037427072.1	TGTGGCACGCCTTTTTCAAG	TGACACGGATCATGCGGAAA
RmSETD4	XM_037425964.1	TTCTTGCCAAACTGCCATGC	TCCTTTAGCAAAGCCCGCAA
RmSETD7	XM_037424082.1	ACCCGAGGTTCAAATTCCGT	TTAGTGGCTGGCTTGTTCGT
RmLSD1-A	XM_037412473.1	TCAACTTCGGCGTGTACGAA	AATGTAGTTGCCCTTGCGGA
RmLSD1-B	XM_037422162.1	ATTCCCTTTGGGGAGTACCA	AGCCATGAAAAGGCGAAGTG
RmGCN5	XM_037436083.1	GCGATCCTCTGCAAGTTGTT	AGCCCTTCCCATGTTTTTCCA
RmDNMT1	XM_037423379.1	TGTTACGTCGTGTATGGCGA	TTTGCCGAGACAACCCATCA
RmDNMT3A	XM_037414329.1	CTGGCGAACATCAACGCAAA	CATGGCAACGATGCTTCCAA
RmDNMT3B	XM_037414367.1	CGTTCCGCACCGTAATTTCA	CGATTTCTCCGCTTCTGCTT
RmMETLL3	XM_037426135.1	GGCAATCAAGTGGAGGGAGTTA	TGGTGGCTTTTTCATGCAGTC
RmMETLL14	XM_037425459.1	TTCCTCTGGTGCGGTTCATC	AGCATATGTCCTCGCATCGG
RmYTHDC	XM_037432474.1	AGCTGTTTCCCGTGGATGAAA	AAATTTTGCTCGCCAGACGC
RmYTHDF	XM_037421839.1	CTGGGCGGTAGTGACAAGAA	CTCCCAAGTGCCAATGTCCA
